# Hyaluronic acid augmentation pharyngoplasty complicated by retropharyngeal abscess and grisel syndrome: Case report and literature review

**DOI:** 10.1002/ccr3.5901

**Published:** 2022-05-20

**Authors:** Abdullah Alkhaldi, Fahad Alwadi, Mazyad Alenezi, Jaber Alshammari

**Affiliations:** ^1^ Otolaryngology Head & Neck Surgery King Abdullah Specialized Children Hospital King Abdulaziz Medical City National Guard Health Affairs Riyadh Saudi Arabia; ^2^ King Abdullah International Medical Research Center Riyadh Saudi Arabia; ^3^ 89660 Department of Otolaryngology Head and Neck Surgery College of Medicine Qassim University Qassim Saudi Arabia

**Keywords:** augmentation pharyngoplasty, grisel syndrome, retropharyngeal abscess, velopharyngeal insufficiency

## Abstract

Augmentation pharyngoplasty, in which tissue filler or grafts are used to augment the posterior nasopharynx, is an accepted option to treat velopharyngeal insufficiency. It is generally well tolerated and safe with limited side effects. In this study, we describe a case of a retropharyngeal abscess and Grisel syndrome following hyaluronic acid augmentation pharyngoplasty. Grisel syndrome is a serious condition that requires early diagnosis and prompt intervention to prevent further complications.

## Abstract 2

Velopharyngeal insufficiency (VPI) can be treated conservatively by speech therapy or surgically by augmentation pharyngoplasty and pharyngeal surgeries. Augmentation pharyngoplasty is safe and well‐tolerated procedure. We report a very rare complication of this procedure which is retropharyngeal abscess and Grisel syndrome (GS). GS is a serious condition that requires early diagnosis and prompt intervention to prevent further neurological complications.

## 1 INTRODUCTION

When the soft palate fails to close against the posterior pharyngeal wall during speech, velopharyngeal insufficiency (VPI) occurs, resulting in hypernasal speech resonance. VPI can be caused by soft palate shortening or functional constraints, as well as the pharyngeal wall musculature on the posterior or lateral sides. Traditional surgical techniques have aimed to extend the soft palate, dynamically tighten the velopharynx, or statically reduce the size of the velopharynx by autologous tissue transfer. Augmentation pharyngoplasty, in which tissue filler or grafts are used to augment the posterior nasopharynx, is an option to treat VPI and has been performed for some time; however, a challenge has been to identify the best filler material that is safe, effective, and provides a predictable degree of augmentation. Recently, it has gained popularity as an option to treat VPI in patients with small to moderate gaps. One potential disadvantage of the use of an injectable filler to treat VPI is durability (i.e., the potential for reabsorption, extrusion, or migration of the implant). It is generally well tolerated and safe, with the most frequent side‐effect obstructive sleep apnea.^1^ Reduced recovery, hospitalization, and anesthesia time are all possible advantages of augmentation pharyngoplasty. We present a rare complication of augmentation pharyngoplasty not yet described in literature. To our knowledge, our report is the first to describe a retropharyngeal abscess and Grisel syndrome following hyaluronic acid augmentation pharyngoplasty. The aim of this paper is to make otolaryngologists aware of this rare condition, which could easily be missed if not suspected early.

## 2 CASE PRESENTATION

A 6‐year‐old girl, medically free, presented to our pediatric otolaryngology clinic complaining only of speech misarticulation. The family denied dysphagia or nasal regurgitation of food/fluids. On examination, the patient had pharyngealization of the fricatives /s/, /z/. The flexible nasoendoscopy showed grade 1 adenoid, intact nasal surface of the soft palate but no movement of the soft palate, and a velopharyngeal gap 2–3 mm with cardinal vowels. The pattern of closure is circular with a Passavant's ridge. Her speech showed a moderate to severe degree of hypernasality, marked imprecision of consonants, nasal grimace, the posterior plosives were replaced by anterior plosives, and nasal emission of air. The speech intelligibility was 2–3/5.

**FIGURE 1:**
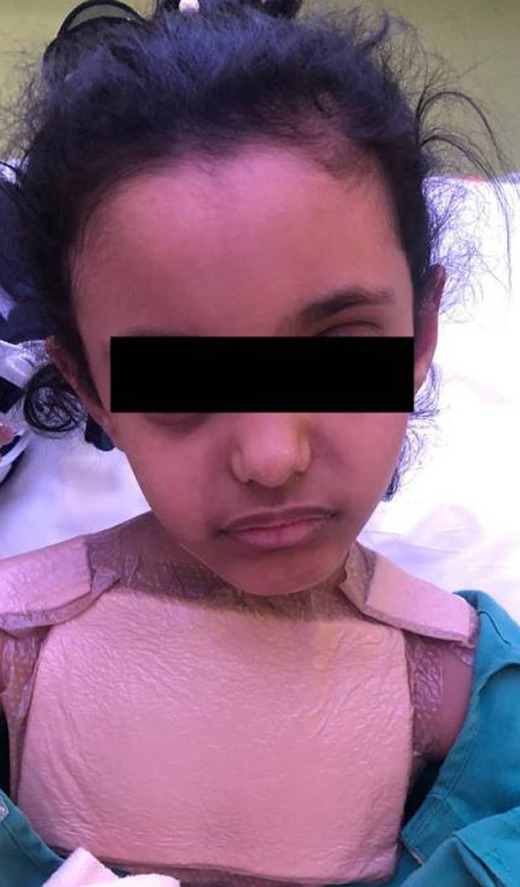
Clinical picture of the patient showing torticollis

**FIGURE 2:**
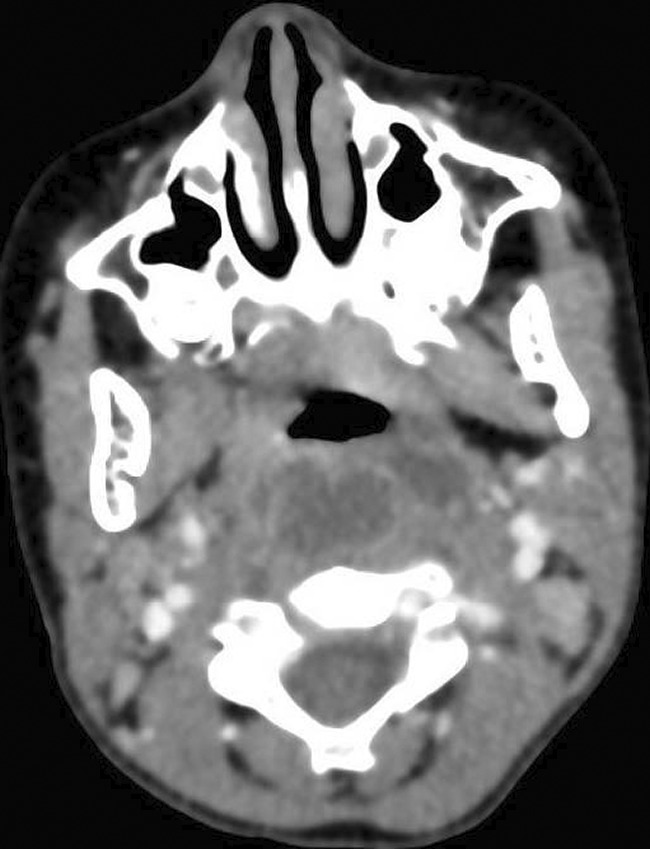
CT head & neck with contrast revealed well‐defined retropharyngeal abscess measuring 3.7 × 1.5 × 2.1 cm

**FIGURE 3:**
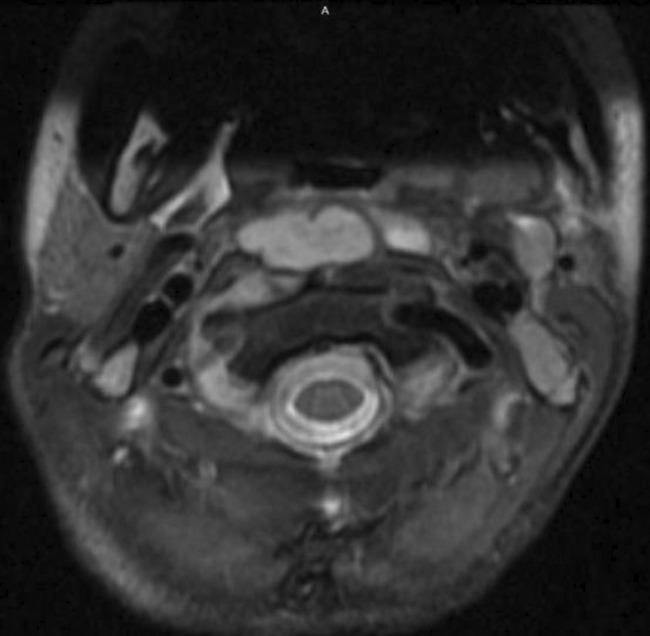
Cervical spine MRI (axial view) well‐defined retropharyngeal/danger space collection measuring 3.8 × 1.4 × 3.1 cm

**FIGURE 4:**
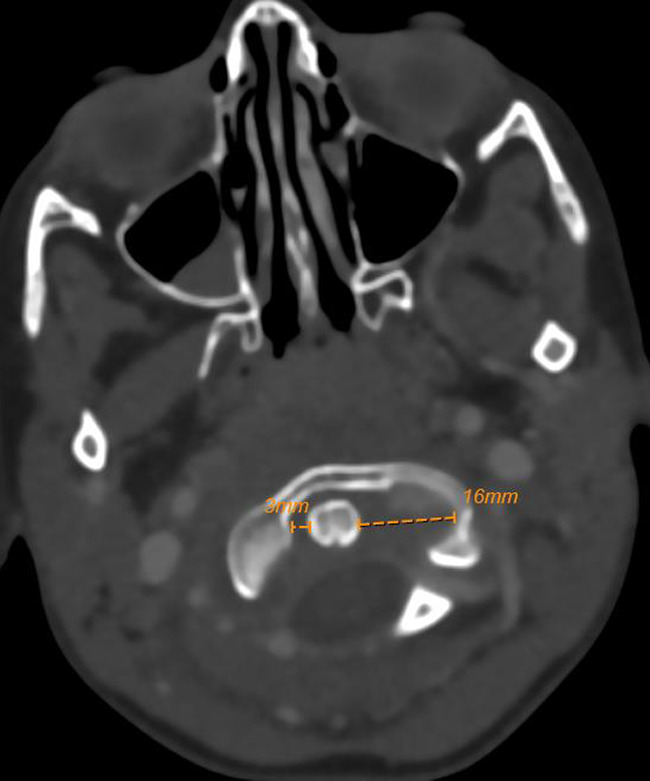
Cervical spine MRI showing mild rotatory atlantoaxial subluxation

**FIGURE 5:**
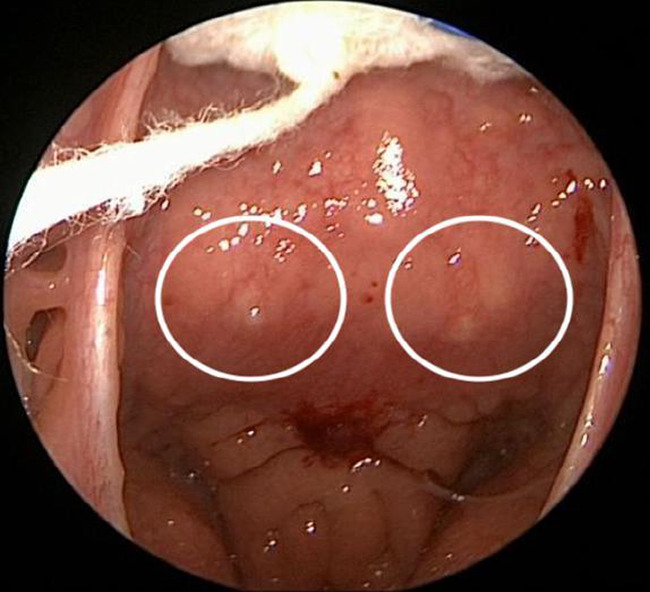
Intraoperative picture showing bilateral retropharyngeal bulging

**FIGURE 6:**
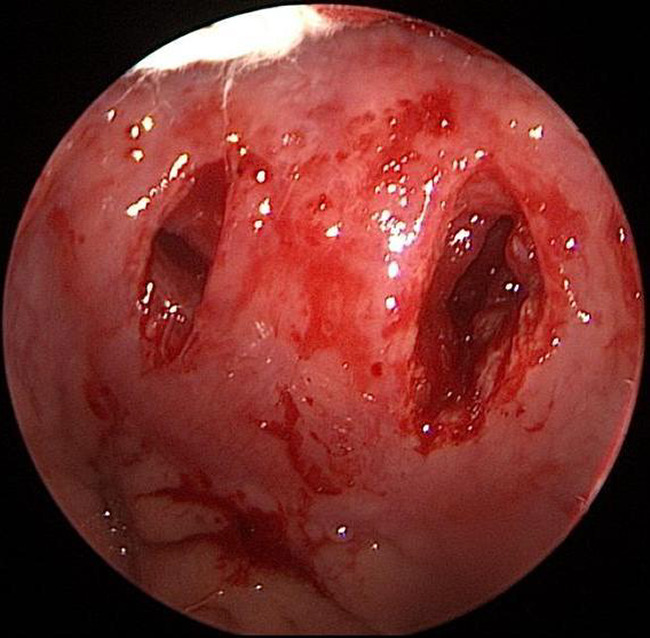
The two retropharyngeal spaces were dissected and kept open

The throat examination showed normal lips, alveolus, and hard palate, a short palate with weak movement, deep pharynx, no overt cleft palate or submucous cleft, and an intact nasal spine. Surgical options were discussed with her family and a shared decision was to start with injection augmentation pharyngoplasty. The risks and benefits of the procedure were explained. After the surgery, the patient was discharged home without any complications. The patient was seen on multiple visits after the surgery; however, the family noticed only a mild improvement in her speech. We offered the family another session of augmentation pharyngoplasty 8 months after first surgery, and they agreed to proceed. In the second augmentation pharyngoplasty, injection of hyaluronic acid was performed with an 18‐gauge needle into the posterior pharyngeal wall (into the retropharyngeal soft tissues). The needle was inserted until it hit the bone, then the needle was retrieved a little, and we injected the material into the median and paramedian areas (a total of 2 ml was used). The second surgery went smoothly, and she was discharged home on analgesics only. Four days later, the patient presented at the emergency department with neck pain, a limited range of motion in her neck, and torticollis (Figure 1). Intravenous antibiotics were started immediately, and she was admitted as a suspicious case of a retropharyngeal abscess. The flexible nasal scope showed a clear nasopharynx with a posterior pharyngeal wall bulge, no discharge was seen, and a patent airway. The CT head and neck with contrast revealed a well‐defined retropharyngeal abscess measuring 3.7 × 1.5 × 2.1 cm with two smaller adjacent collections (Figure 2). The cervical spine MRI showed a well‐defined retropharyngeal/danger space collection (measuring 3.8 × 1.4 × 3.1 with a volume measuring 9 ml) with extensive inflammatory changes extending to the epidural space and mild rotatory atlantoaxial subluxation (Figures 3 and 4). The neurosurgery team was consulted, and a C‐Collar was applied to the patient with no need for a surgical intervention for the mild rotatory atlantoaxial subluxation. Patient was diagnosed with a retropharyngeal abscess, complicated by Grisel syndrome (GS). The patient was prepared for a surgical incision and drainage of the retropharyngeal abscess under general anesthesia. She was intubated with no difficulty. Standard intubation precautions of retropharyngeal abscess were taken. The patient was cleared by neurosurgery for any cervical instability related to Grisel syndrome. The patient was positioned in a Trendelenburg with a tonsil ball around the endotracheal tube to prevent aspiration of the abscess in the airway. The intraoperative finding showed bilateral retropharyngeal bulging, more on the right side (Figure 5). Two paramedian vertical incisions were made bilaterally at the bulging sites and extended for 2 cm. The abscess was collected by a suction trap (7 ml was suctioned) and sent for microbiological and histopathological analysis. The two retropharyngeal spaces were dissected and opened to each other by a curved artery forceps (Figure 6). Both wounds were irrigated with diluted povidone followed by saline irrigation and kept open. The patient was extubated with no airway issues and transferred to a regular ward. After the surgery, the patient's status improved substantially and she resumed a soft diet for the first three days, then a regular diet. The patient continued with the IV antibiotics and the C‐Collar. Day five post‐surgery, she was discharged home in a stable condition on oral Amoxicillin/Clavulanic acid and the C‐collar. During the follow‐up period, she was doing fine, no pain, tolerating oral feeding very well, and there was a significant improvement in the head position. The second augmentation pharyngoplasty was different because a larger caliber needle was used, injection of hyaluronic acid was deep, and the patient was not discharged on antibiotics as the first surgery.

Precautions to be considered (to avoid this kind of complications) ^1^: A small caliber needle should be used (25‐gauge needle). ^2^ Injection should be superficial at the level of pharyngeal constrictor muscles and para‐vertebral fascia targeting retropharyngeal soft tissue.^3^ Volume should not exceed 4 ml.(4) Patients should be discharged on antibiotics after surgery.

## 3 DISCUSSION

Traditional techniques to treat VPI surgically are safe and well‐tolerated. Complications related to augmentation pharyngoplasty were recently reported by Cofer et al. In a review with 50 patients who underwent injection pharyngoplasty for VPI, the most frequent complication was snoring (16%), followed by severe neck pain (14%). ^1^

Grisel syndrome (atlantoaxial subluxation) was first described by Charles Bell in 1830; however, it was named after Grisel who reported two cases in 1930.^2^ It refers to atlantoaxial rotatory subluxation not associated with trauma or bone lesion. It is has been linked to infections of the upper airway (pharyngitis and mastoiditis), head, and neck region (retropharyngeal abscess), or with otolaryngology surgeries such as adenoidectomy.^3^, ^4^ A systematic review conducted by Karkos et al. indicated that GS is associated with surgical interventions of the head and neck region (40%), with adenotonsillectomy being the most frequent (78%). Less frequently, it also occurs after procedures such as pharyngoplasty (2.5%) and otoplasty (2.5%). ^5^ Spontaneous subluxation due to inflammatory conditions of the head and neck region is rarely encountered in routine otolaryngology practice; however, it is a serious condition that requires early diagnosis and prompt intervention to prevent neurological sequelae.

The incidence rate of GS is not known yet; however, it is frequently seen in the pediatric age group, which could be due to the relatively larger head size, loose ligaments and joints, and a more horizontally placed facet joint. The greater number of retropharyngeal lymph nodes and the rich lymphatic system may potentially contribute to the higher incidence of the disease in the pediatric population.^6^

The pathophysiology of atlantoaxial subluxation is not well‐known. It is considered that the subluxation takes place because of laxity of the ligaments around the atlantoaxial joint. Hyperemia from the hematogenous spread of the infection through the pharyngovertebral veins causes this laxity.^3^ There is no pathogen that has been identified in the pathogenesis of this disease.

Patients with GS typically present with an abnormal head tilt. This abnormal head tilt has been classically described as a Cock Robin tilt where the chin is rotated to one side while the neck is flexed to the opposite side. A painful palpable C2 spinous process indicates subluxation.^7^

The diagnosis of GS should be based on a high index of suspicion, in every patient who presents with painful torticollis after upper airway or head and neck infection, or after ENT surgery. The diagnosis depends mainly on clinical and radiologic findings. The diagnostic workup requires a complete neuroradiological examination, including plain X‐rays, CT scan, and MRI. CT and MRI are both excellent diagnostic tools to evaluate deep neck infections and the condition of the bones and the ligaments of the cervical spine.^5^ However; the gold standard diagnostic tool of atlantoaxial rotary subluxation is a CT scan with three‐dimensional reconstruction which evaluates the atlanto‐dens interval, supporting classification according to the Fielding–Hawkins grading system (Table 1). ^8^, ^9^ It should be suspected in patients presenting with fever, painful torticollis, restricted range of motion in the absence of trauma, but with a history of upper airway or head and neck infections, or otolaryngology surgery.^10^, ^11^

**TABLE 1:** Fielding–Hawkins classification^9^

Type 1	Rotation of the atlas on the axis without displacement, or with anterior displacement ≤3 mm.
Type 2	Rotatory fixation with anterior displacement of the atlas 3–5 mm.
Type 3	Rotatory fixation with anterior displacement of the atlas ≥5 mm.
Type 4	Rotatory fixation with posterior displacement (extremely rare condition).

There is no gold standard management for this condition, even though various recommendations have been proposed. There is consensus on the use of progressively more invasive treatment according to the Fielding–Hawkins grade (Table 1). Most of the cases included in a systematic review by Karkos et al. responded well to conservative management including antibiotics, anti‐inflammatory drugs, bedrest, muscle relaxants, cervical traction, and external immobilization using a soft or hard collar. Early broad‐spectrum antibiotics are necessary to prevent the spread of the infection.^5^ In short, treatment should be individualized. Type I and II may need more aggressive treatment in case of neurological complications or failure of conservative treatment.

## 4 CONCLUSION

GS is a condition which is unrecognized and usually missed at the initial presentation, especially when associated with ENT surgery. It is a serious condition that requires early diagnosis and prompt intervention to prevent further neurological complications. In this study, we described a case of retropharyngeal abscess and Grisel syndrome following hyaluronic acid augmentation pharyngoplasty, which was successfully managed without further complications.

## AUTHOR CONTRIBUTIONS

Abdullah Alkhaldi: Data collection, Manuscript writing, and Manuscript review. Fahad Alwadi: Data collection, Manuscript writing, and Manuscript review. Mazyad Alenezi: Manuscript writing and Manuscript review. Jaber Alshammari: Manuscript writing and Manuscript review.

## CONFLICT OF INTEREST

The authors have no relevant financial or non‐financial interests to disclose.

### ETHICAL APPROVAL

This paper was reviewed and ethically approved by King Abdullah International Medical Research Center, Riyadh, Saudi Arabia. This research involves human participant(s) and/or animal(s).

### CONSENT

Written informed consent was obtained from the patient to publish this report in accordance with the journal's patient consent policy.

## Data Availability

The data used to support the findings of this study are available from the corresponding author upon request.
